# Circular Economy Transition in an Emerging Economy: Current Status and Priorities in Peru

**DOI:** 10.1007/s43615-026-00791-9

**Published:** 2026-03-02

**Authors:** Alejandro Gallego-Schmid, Ricardo Rebolledo-Leiva, Leonardo Vásquez-Ibarra, Alvaro Elorrieta-Mendoza, Denisse Milagros Paredes Cotohuanca, Claudia E. Henninger, Ana Belén Guerrero

**Affiliations:** 1 Tyndall Centre for Climate Change Research (Manchester), Department of Civil Engineering and Management, School of Engineering, University of Manchester, Booth Street E, Manchester, M13 9PL UK; 2 Department of Computing and Industries, Faculty of Engineering Sciences, Universidad Católica del Maule, Av. San Miguel 3605, Talca, Chile; 3 Centro de Innovación en Ingeniería Aplicada (CIIA), Faculty of Engineering Sciences, Universidad Católica del Maule, Av. San Miguel 3605, Talca, Chile; 4 Peruvian Life Cycle Assessment & Industrial Ecology Network (PELCAN), Departamento de Ingeniería, Pontificia Universidad Católica del Perú, Av. Universitaria 1801, San Miguel, Lima, 15074 Peru; 5 Departamento de Ingeniería, Pontificia Universidad Católica del Perú, Av. Universitaria 1801, San Miguel, Lima, 15074 Peru; 6 Department of Materials, School of Natural Sciences, University of Manchester, Booth Street E, Manchester, M13 9PL UK; 7 Instituto de Investigación en Ciencias Naturales y Tecnología (IARNA), Universidad Rafael Landívar, Vista Hermosa III, zona 16 , Ciudad de Guatemala, Guatemala; 8 Trisquel Consulting Group, Quito, Ecuador

**Keywords:** Global South, Public policy, Environmental sustainability, Local economies, Informal recycling, Waste management, Bioeconomy

## Abstract

The circular economy is increasingly recognised as a multidimensional paradigm that goes beyond reducing waste and recycling, encompassing systemic design, regenerative practices, socio-technical innovation, and equity considerations. This study examines Peru’s efforts to transition towards circularity as a representative emerging economy in Latin America, based on 15 semi-structured interviews with stakeholders from academia, policymaking, business, and non-governmental organisations. It provides new empirical insights by identifying context-specific drivers and barriers not previously reported in the literature. The analysis identifies barriers such as limited consumer awareness, weak enforcement of regulations, technological gaps, and financial constraints affecting especially micro, small, and medium enterprises. Drivers and strengths include international cooperation, biodiversity, ancestral knowledge, and emerging policies. The government, European agencies, and civil society emerge as central actors in this transition. Conceptually, the study offers a novel contribution by adapting Schlossberg’s transition theory, traditionally applied in psychology and education, to the field of sustainability. Findings emphasise the need to raise awareness, foster a cross-sectoral and decentralised regulatory framework with clear responsibilities, enforcement, and accountability mechanisms, promote targeted investments, and design policies that integrate informal sectors. This research contributes to both theory and practice: methodologically by extending transition theory to sustainability studies, and empirically by providing the first multi-stakeholder analysis of CE in Peru, offering insights for policymakers seeking to balance environmental and economic goals.

**Supplementary Information:**

The online version contains supplementary material available at https://doi.org/10.1007/s43615-026-00791-9.

## Introduction

 The circular economy (CE) is a systemic paradigm, restorative and regenerative by design [1–3], that seeks to transform production and consumption models by replacing the notion of “end-of-life” with that of a continuously valorised resource [2, 4]. In contrast to the linear “take–make–dispose” model, the CE advances the slowing, closing, and narrowing of material and energy loops, thereby minimizing virgin resource extraction, emissions, and energy leakages. Its purpose is to preserve the value of products, components, and materials through strategies such as eco-design, repair, reuse, remanufacturing, and recycling. The CE operates across multiple scales—micro (products, firms, consumers), meso (eco-industrial parks), and macro (cities, regions, nations)—and relies on the joint action of businesses, consumers, policymakers, academia, and technological innovation as key enablers. Ultimately, the CE aims to decouple economic growth from resource pressure, simultaneously generating environmental quality, economic development, and social equity for the benefit of present and future generations [1, 3].

Despite the growing academic interest in CE transitions in the Global South, research remains fragmented and often limited to descriptive or sector-specific studies. For example, sectoral case studies, such as those in Malaysia’s manufacturing industry [5] and Namibia’s mining sector [6], have deepened the understanding of local barriers and enablers, yet highlight the absence of inclusive, systemic perspectives that account for diverse stakeholders. On the other hand, recent reviews have synthesised CE practices in low- and middle-income contexts [7], examined regional dynamics in Latin America using structured frameworks such as PESTLE [8], and critically compared CE policy trajectories between the Global North and South [9]. However, these studies collectively underscore a key concern: the risk of uncritically replicating Global North approaches in contexts with distinct socio-political and institutional realities. The Latin America and the Caribbean region (LAC) has a high level of interest in the circular model, by publishing over 80 public initiatives [10] Colombia was the first country in the region to publish a National Circular Economy Strategy in 2019 [11] followed by Chile in 2021 [12], and Uruguay in 2024 [13]. Recently, Perú has launched its national strategy [14] in convergence with regional patterns. However, existing literature focused on Peru has explored CE initiatives in areas such as plastics regulation [15], the water management sector [16], and business adoption of CE practices [15, 17]. Similarly, Ospina-Mateus et al. (2023) identified 25 CE-related studies in Peru [18], revealing a focus on isolated sectors rather than a holistic assessment of national CE dynamics. This demonstrates an enterprise-level attention, with limited insights into the roles of civil society, policymakers, and institutional frameworks.

In the Global South, the influence of informality on circular systems has been predominantly analysed within the waste management sector [19]. Empirical studies highlight diverse cases: plastic waste recycling in Kenya, where economic incentives, trust, and adaptive capacity are critical to sustaining supplier–buyer relationships [20]; the characterization of e-waste pickers in South Africa [21]; the identification of key dimensions of informality among e-waste pickers in Chile—namely agility (speed of income generation), capillarity (extent of material collection), and flexibility [22]; the systematic undervaluation of the expertise and operational efficiency of informal waste pickers and non-profit organizations in waste sorting and processing [23], and the potential framing of informal recyclers’ formalization within the circular economy (in Argentina [24]).

## Theory

This article draws on transition theory, thereby exploring the shifts within a country and more broadly society. In the past, studies have investigated individual aspects of the transitioning process by examining demographic transitions (e.g., birth/death rates) and their impact [25], economic transitions, predominantly from a planned to a market economy [26], or sustainable transitions. The latter has been researched within the Global North and seeks to promote sustainability through socio-technical transformations [27]. Whilst instructive, past research has focused more on individual aspects within society, whether this be population growth rates or popularising sustainability measures. What is currently lacking is to understand how individual players are supporting these transitions within a country. Thus, this article examines transition theory through a psychology lens by critically adapting Schlossberg’s 4Ss model [28], originally developed to understand how individuals experience and respond to life transitions, to examine the systemic shift towards a CE in Peru. While Schlossberg’s framework is traditionally applied to personal transitions (e.g [29, 30]), we argue that some of its conceptual elements can offer valuable insights into how collective entities, such as nations, navigate complex and multifactorial changes by focusing on individual players within these nations. However, this adaptation is not direct nor without caveats; rather than equating individuals with economies, the model is employed to explore the multi-dimensional dynamics shaping the CE transition in a context marked by institutional fragility, economic informality, and social inequality. As such, the model is used as a tool to guide the researchers in exploring the dynamics within Peru and how these dynamics are shaping CE practices.

The model identifies three types of transitions: (1) anticipated, (2) unanticipated, and (3) non-events, which serve as entry points for interpreting Peru’s CE process and guide the understanding of levels of power within the country. The CE shift can be seen as anticipated, given growing policy and business interest; yet it is also shaped by unanticipated events such as the COVID-19 pandemic, global supply chain disruptions, or climate-induced disasters. Additionally, non-events, such as the failure to implement draft legislation, highlight barriers where planned transitions fail to materialise due to lack of political consensus or enforcement capacity. This is explained in detail in Sect. 4.

Beyond classifying transition types, the model’s core analytical tool (the 4Ss: Situation, Self, Support, Strategy) has been selectively adapted to reflect institutional and systemic conditions. For example:

- *Situation*: is interpreted as the structural and institutional context in which the CE transition occurs, including political stability, regulatory frameworks, and socio-cultural norms.
- *Self*: is reconceptualised to reflect Peru’s internal capacities for change, such as sectoral readiness, infrastructural limitations, or public engagement.
- *Support*: encompasses the financial, legislative, and technical mechanisms available to facilitate or hinder CE implementation.
- *Strategy*: refers to national and sectoral pathways for operationalising CE principles, including regulatory instruments, voluntary agreements, and educational campaigns.

While recognising the limitations of applying a model designed for individual change to national policy transitions, this article treats the framework as a new lens to structure the analysis of transition dynamics. The contribution lies in showing how this adapted perspective can reveal interactions between institutional capacity, external shocks, and strategic responses that shape the trajectory of CE implementation in a Global South context. To the best of the authors’ knowledge, this is among the first attempts to interpret national CE transitions through the lens of a psychosocial transition model, opening new avenues for interdisciplinary dialogue and the development of context-sensitive analytical tools.

Building on the theoretical foundations outlined above, this study aims to provide one of the first integrated, context-sensitive analyses of the drivers, barriers, and stakeholders shaping the CE transition in Peru. Drawing on semi-structured interviews with local experts, it offers new conceptual and empirical insights into how the CE is understood and implemented in an emerging economy, applying Schlossberg’s transition theory to examine the interplay of political, cultural, and economic factors in the transition process. In addition, it highlights key priorities and recommended actions identified by local experts, providing valuable guidance for decision-makers to support the transition to a circular economy, as well as power dynamics.

## Methodology

Before data collection, a research protocol, based on previous studies [31–33], was designed to guide the interview process (see Online Resources for full details). Between March and May 2024, a total of 15 in-depth semi-structured interviews were conducted with key stakeholders involved in areas surrounding the CE in Peru, categorised into four primary groups [33, 34]: academia, policymakers, businesses, and non-governmental organisations (NGOs) (Table 1). To ensure a comprehensive perspective, representatives were drawn from different levels (e.g. national, regional, and municipal governance and from micro, small, and medium-sized enterprises (MSMEs) to large companies). Semi-structured interviews were conducted as these allowed for a predetermined set of open-ended questions, while also offering the flexibility to go deeper into relevant topics through a conversational approach [35, 36]. This method enabled interviewees to provide detailed insights into their experiences and the challenges they faced while engaging with CE initiatives in Peru.

**Table 1:** Profile of the interviewees and duration of the interviews

Group/Abbreviation	Profile	Duration
Academics		
A1	Professor (circular electronic waste and mining)	37:59
A2	Lecturer (circular fishing and aquiculture)	59:29
A3	Lecturer (circular plastics in the oceans)	38:08
A4	Lecturer (circular mining and procurement)	1:26:07
**Policymakers**		
P1	Senior position in the Ministry of Environmental Management	1:07:49
P2	Main contributor to the National Economic Roadmap for Peru proposal	58:08
P3	Senior position in the Ministry of Environmental Protection and the Ministry of Production	1:10:41
**Businesses**		
B1	Sustainability and research head in a fishing company	1:34:51
B2	Senior official at the Lima Chamber of Commerce	1:11:33
B3	Head of a company assessing the reduction of plastic use	1:10:05
**Non-governmental organisations (NGOs)**		
N1	Circular economy consultant working with NGOs	1:05:26
N2	Leader of a social enterprise specialised in circular food waste	52:31
N3	Circular economy consultant working with NGOs	1:48:43
N4	Specialist in circular and sustainable finance, integrated plastic management and cleaner production working with NGOs	1:42:27
N5	Specialist in solid waste management working with NGOs	53:19

A combination of purposive and snowball sampling was utilised to ensure participants were able to respond to the questions [26]. The initial selection of participants was guided by their expertise related to knowledge or practical involvement with CE-related activities in Peru (e.g., policy design, project implementation, or academic research). Additional participants were identified through snowballing, as proposed by the previous interviewees. The profiles of the proposed candidates were analysed to see that they cover new CE perspectives not considered before. This interdisciplinary and multi‑sectoral selection ensures that the collected data captures the complexity of the system. Data collection continued until thematic saturation was achieved, defined as the point at which no new codes or themes emerged across three consecutive interviews. Saturation was reached after 15 interviews, confirming that the sample size was sufficient for capturing the breadth of perspectives required.

Regarding the number of interviews, in qualitative research, the adequacy of a sample is judged by data saturation (the point when no new themes emerge) and information power (the richness and relevance of the data), not by numbers alone. Methodological studies show that most themes appear within the first 10–15 interviews. For example, Guest et al. (2006) found saturation by 12 [37], while Hennink & Kaiser (2021) reported 9–17 as typical [38]. Leading methodologists like Creswell (2013) suggest ranges of 5–25 interviews for in-depth studies [39]. When participants are experts or key stakeholders, each interview tends to be information-rich, meaning fewer are required [40]. Thus, 15 well-targeted, in-depth interviews with experts across multiple sectors can be entirely sufficient for robust national-level insights. In addition, there are also strong precedents in sustainability and policy research studies on circular economy, housing, and energy policy, which have all used around 15 interviews to produce credible national analyses. For instance, a 2021 study in Mexico on circular economy in housing relied on 15 interviews with experts across government, business, and NGOs [41], while other sustainability studies reported reaching saturation after about a dozen interviews [42, 43]n such cases, what matters is that all relevant stakeholder perspectives are represented and themes become repetitive, showing that additional interviews would add little. Accordingly, in this study, 15 interviews are not a limitation but a methodologically sound design choice supported by both theory and precedent.

Interviews were conducted in Spanish, either face-to-face or online, and were recorded, transcribed verbatim and translated into English using a back-translation technique. The latter ensures that meaning loss in the translation process is minimised. To enhance the robustness of the findings, interviews were complemented with document analysis, including policy roadmaps (e.g [14]). , laws (e.g [44]). , and regulatory frameworks (e.g [45]). related to CE in Peru, as well as secondary academic and grey literature (e.g [46]). These sources were used to triangulate interview data, corroborate stakeholder perspectives, and situate the results within broader national and regional policy developments. This triangulation with secondary sources reduced reliance on a single dataset, reducing potential biases. Even more, the multi-sectoral design helped avoid dominance of one stakeholder group and peer debriefing within the research team ensured that emerging interpretations were challenged and refined. Finally, anonymity was guaranteed to participants, reducing social desirability bias and encouraging candid responses. This anonymity was part of the ethical standards the study adhered to, with necessary approvals obtained before data collection (Ref no.: 2024-18271-32729). Participation was entirely voluntary, with informed consent secured from all participants.

The analysis followed Easterby-Smith et al.’s (2012) seven-step framework, starting with familiarisation and reflection, then moving to conceptualisation, coding, linking, and re-evaluation. An initial set of a priori codes (barriers, drivers, strengths, opportunities, stakeholders, priorities, specific aspects for Peru and practical examples) was developed from the literature and the research protocol. This was iteratively refined through in vivo coding to capture participants’ own terms and perspectives. Codes were then grouped into broader themes during several rounds of recoding. To ensure reliability, two members of the research team independently coded a subset of transcripts and reconciled discrepancies through discussion until consensus was reached. The final coding structure was applied consistently by the lead author. Validity was reinforced by team discussions of emerging interpretations, peer debriefing sessions within the research group, and consistency checks between transcripts, codes, and themes.

## Results and Discussion

To provide a comprehensive perspective of the CE in Peru, the main barriers, drivers and strengths (Table 2), as well as key stakeholders and priorities identified from semi-structured interviews, are analysed in detail in the following subsections.

**Table 2:** Barriers, drivers and strengths for circular economy implementation in Peru

Category	Barriers	Drivers	Strengths
**Culture**,** education**,** and society**	- Consumerist mentality and preference for cheaper over circular products. - Lack of specific education and professional training.	- Formalisation of labour in the waste management sector. - Citizen awareness is emerging based on the effects of climate change in the country.	- Peru’s biodiversity and ancestral cultures create a solid basis for the circular economy.
**Policy and Regulation**	- Laws without strong enforcement. - Regulatory inconsistency and lack of coordination among government and private stakeholders. - International policies not adequately tailored to the local context.	- The political will to advance the circular economy. - International cooperation, mainly from the European Union. - Cleaner production agreements.	- A multi-stakeholder coalition at a national level.- - Circular economy roadmap.
**Technology and Data**	- Lack of investment in academic research. - Technological and infrastructural deficiencies and unevenly distributed.	- Research focused on recycling technologies and waste management. - Cooperation with international universities.	- Industry empowerment through innovative sustainable practices, such as eco-industrial parks.
**Economy**	- Small companies perceive circular practices as costly. - Reliance on resource-intensive industries with an undeveloped recycling industry. - Lack of economic or fiscal incentives to foster the transition. - Insufficient and inconsistently allocated funding.	- Large international companies are introducing circularity in their operations. - Emerging financial options from the public and private sectors. - Motivation of companies to differentiate themselves and protect their reputation. - International market pressures (e.g., certifications)	- Empowerment of the industrial sector, encompassing both small, medium and large companies.

### Barriers

#### Culture, Education and Society

Focusing on the *situation* [28] first, the aptitude for change can be explored through investigating cultural aspects. Despite Peru’s long tradition of repair and reuse, influences from affluent, consumption-driven societies like the United States, which favour new over repaired or recycled products, often undermine these circular practices (A1; N3; N5). Socioeconomically, lower-income communities (30% in poverty and 5% in extreme poverty [47]) lack the resources and support to engage in CE initiatives (A4; B3; N4; P3). Consequently, most consumers prioritise price, choosing cheaper over circular products, which are seen as elitist and costly (A2; A3; A4; B2; P2; P3; N2). Additionally, cultural resistance to change and entrenched linear economic practices limit consumer engagement and demand for circular products and practices (A1; A2; A3; B3; P1; P2; N3; N5). This is further evidenced by national data showing that only 22.6% of Peruvians mention sustainability among their main interests, with figures dropping sharply in lower-income sectors [48]. This cultural barrier is consistent with broader patterns observed across Latin America, where excessive consumerism and a low level of environmental awareness have been identified as critical obstacles to CE adoption [49–51].

Most of the interviewees agree that a major barrier lies in Peru’s educational system, which inadequately incorporates CE principles into its curriculum. However, a reduced number of interviewees highlighted some initial steps like the recent creation of CE-related courses and diplomas, as well as the integration of CE concepts into undergraduate programs (A4; B3; P1; P3), signalling a curriculum update (N5; P1). Additionally, there is a lack of targeted professional training for government officials, businesses, and the public, especially among older generations (Generation X and above). This deficiency leads to a workforce unprepared to implement and manage CE practices. Consequently, the public has a limited understanding of what a CE is, and public officials and decision-makers exhibit significant knowledge gaps. This lack of comprehension hinders the creation of suitable policies, grassroot initiatives, market demand for circular products, and necessary investments for transitioning to a CE (A2; A4; B1; B2; N2). Furthermore, even among those familiar with the term, many associate a CE only with recycling, often seen as a low-status activity (A2; A3; B3; N1; N4; N5; P2), which impedes the adoption of CE principles at the community level (N2). This situation mirrors challenges across the Latin American region, where low education levels, a lack of environmental communication from governments, and undervaluation of informal knowledge and workers have been widely reported [8, 18].

Reflecting on transition theory, it becomes apparent that although traditionally Peruvian culture supports CE practices, as aspects such as repair and reuse are ingrained in what they do as a society, globalisation has shifted this thinking. With individuals preferring to buy new and starting to avoid stigmatisation through reuse, practices that are seemingly vanishing in support of materialism. In linking this to *support*, it becomes evident that CE practices are currently not fully supported by educational institutions, which can further slowdown and/or prevent transitioning, as individuals may be unaware of the consequences and/or benefits of a CE. Furthermore, as Jaeger-Erben et al. (2021) argue, addressing equity, inclusion, and social justice is essential to ensure CE transitions are not only technically effective but also socially legitimate [52].

#### Policy and Regulation

All interviewees agree that since 2016, Peru has rapidly increased CE regulations, but the typical approach of enacting laws without strong enforcement undermines their effectiveness. A4 illustrates this by saying, “*we have many traffic lights*,* but not enough police to verify that people respect the traffic light.*” This ineffective enforcement, especially among small and informal businesses, leads to poor compliance and greenwashing, where measures are superficially adopted without real environmental benefits (N1; N4). Recent evidence supports this perception: for instance, the Ombudsman’s Office has warned that, seven years after the enactment of the Single-Use Plastic Law (Law 30884), several technical regulations are still pending, which limits the effectiveness of enforcement and compliance [53] Additionally, regulatory confusion, inconsistency, and lack of coordination among government entities and private stakeholders exacerbate the issue, as multiple agencies independently issue fragmented, sector-specific, and inefficient regulations (A2; B2; N1; N4). Stakeholders may benefit from these strategies, but are unlikely to adopt them due to time, financial, or knowledge constraints. This links to what Schlossberg (2011) terms the *self*, as organisations are enforcing a coping mechanism, in which they get by with only implementing what is necessary, but without going the extra mile (reactive strategy). Activating this coping mechanism implies that stakeholders comply with what is necessary, but overall, the transition is slowed down and, in the worst-case scenario, even hindered. This lack of strong and enforceable regulations is a persistent challenge in the Latin American region as a whole, particularly in the area of solid waste management [8, 18]. As in Peru, across LAC countries, regulatory frameworks often lack coherence and continuity, with policy initiatives frequently disrupted by institutional instability and short political cycles.

The drive towards a CE in Peru is significantly influenced by global trends and international organisations (A1; A4; B1). However, this external influence often leads to policies that are not adequately tailored to the local context, causing implementation challenges, especially when laws are copied from other regions without adaptation to Peru’s realities (A4; B1) “*Replicating policies from Europe here do not work in many cases*” (B1). Most interviewees highlight the crucial role of local governments in waste management and recycling at the national level, but note a frequent disconnect between national policies and local implementation, neglecting regional specificities and needs, which creates inconsistencies and inefficiencies. Since 2017, Peru has faced political instability with six different presidents. All interviewees agreed that internal rivalries and personalistic approaches within ministries hindered the continuity and commitment to long-term CE initiatives, worsened by high turnover rates among public officials. This instability fosters scepticism and reduced stakeholder engagement, with N2 stating, “*unfortunately*,* in Peru*,* the State is so degraded that we have lost faith in it.*” Political willingness is also essential; without government prioritisation of environmental issues, implementing effective CE policies is challenging (N1; P1). This is exacerbated in Peru by corruption and the influence of powerful industry lobbies, which obstruct CE progress, with enforcement actions often used for extortion rather than ensuring compliance.

Here, the interplay between all 4Ss can be observed, in that the political instability (*situation*) can have an impact on the *support* that is available to companies to act upon, making changes. To comply with international laws and be able to continue business outside the national boundaries, reactive approaches are implemented. From the data set, it seems that economic benefits (especially monetary) can dramatically alter the course and thus are seen as key drivers (see Sect. "Drivers").

#### Technology, Research and Data

Interviewees agree that, although recent efforts to advance research addressing CE aspects in Peru have increased, much of the work remains theoretical, outdated, and fragmented. This impedes comparative analysis and contributes to a relatively low level of research focusing on a CE in Peru compared to other Latin American countries (N2; N3; P1; P2; P3). Most interviewees affirm that a historical lack of investment in academic research has hindered innovation, resulting in a sluggish transition from theoretical concepts to practical applications. A weak link between the private sector and academic institutions exacerbates this issue further, as limited collaborations and investment obstruct the implementation of research findings into actionable strategies (A3; P3; N3).

The interviewees concur that technological and infrastructural deficiencies significantly hinder CE practices in Peru. High costs of importing advanced technologies, inadequate financial incentives, and limited awareness about the benefits of CE practices restrict adoption, especially among MSMEs. “A*n investment of this type [in a MSME] has to be thought through carefully*” (N5). Additionally, technological infrastructure is unevenly distributed, with rural areas and regions like the Amazon and Andes experiencing poor connectivity and limited access to modern technologies (P3; A2; B3; N3; B1). Waste treatment infrastructure is also inadequate, being heavily centralised in Lima, which results in insufficient landfills and recycling facilities in other regions, causing significant environmental and health issues (A2; A3; B1; B3; N2; N3; N5; P1; P2). The lack of a robust system for managing waste and secondary materials obstructs effective recycling, industrial symbiosis, and material recovery (A2; A3; B1; B3; N1; N2; P1; P2). Current international funding, mainly from entities like the World Bank, focuses on training rather than building the necessary infrastructure to support a CE. “*It is crucial to improve the waste management system in the country to improve this migration towards the circular economy*” (A2). Moreover, the pervasive informality in the business sector and limited mechanisms for information sharing and collaboration among stakeholders complicate data collection and traceability (A2; N3; N4; N5; P1). This hampers the development of comprehensive CE strategies and the assessment of their benefits, making initiatives seem like cost burdens instead of investments. Standardised metrics, effective indicators, and better data availability are essential for measuring progress, identifying improvements, informing policy decisions, and promoting CE practices nationwide (A1; P1; N3; N4; N5).

A lack of infrastructure (*self*) and *support* can be seen to impact the transition process, in that strategies need to be implemented that can foster the transition process. This highlights that the transition overall may be possible, but will be slow, as current structures available in the country may not fully support the process (e.g., lack of access in the Amazon and the Andes).

#### Economy

MSMEs are crucial in Peru, representing 99% of the economy [54] and over 70% of the workforce in the informal sector [55]. However, many lack awareness of CE benefits and the tools to engage effectively (A2; A4; N3; N5). Most MSMEs view CE measures as costly expenses rather than investments, prioritising short-term profits (A2; A4; B1; N2; N3; P2). *“In many cases [companies] are informal and then informality is often associated with environmental deterioration because there are no management systems implemented*,* resources or knowledge*” (N3). Formal companies typically comply only with minimum regulatory requirements to avoid penalties, with few adopting voluntary measures unless driven by certification or stringent laws (A2; A3; A4; N3; P2). Additionally, the underdeveloped market for recycled and upcycled products faces heavy competition from imported virgin materials, limiting demand and profitability (A1; A2; A4; B1; N1; N3). Peru’s reliance on resource-intensive industries like mining and agriculture, which follow linear models, also poses significant challenges to CE adoption (A2).

All interviewees agree that financial challenges significantly hinder Peru’s transition to a CE. The lack of robust public fiscal policies to discourage wasteful practices or incentivise recycling and reuse exacerbates this issue. In terms of private funding, banks offer limited support for CE projects due to perceived high risks and uncertain returns, especially for informal companies, restricting the scaling and professionalisation of CE efforts (A4; N3). Although some international and local funding initiatives exist, they are insufficient and inconsistently allocated (A1; A4; N4; N5; P1). P1 stated, “*there are no tax incentives*,* no financial incentives*,* no type of incentives other than honorary incentives. Many companies genuinely want to engage in CE activities but simply lack the time and resources to do so.*” MSMEs aware of CE benefits often struggle to secure funding due to limited and inadequate public financial incentives, which are typically burdened by cumbersome administrative and legal requirements (A2; A4; N3; N4; N5; P3). According to IDB Invest (2024), financing for circular investments in Peru faces key barriers: the absence of a common taxonomy, banks’ perception of high risk, limited financial products adapted to MSMEs, and the need for blended finance instruments and categorisation systems to mobilise private capital [56]. Furthermore, most MSMEs involved in recycling and the CE operate within the informal economy, lacking resources for scaling up, relying on non-technological procedures, and having short lifespans (A1; A2; A4; B2; N3; N4; N5). These factors complicate the integration of circular activities into formal strategies and create unfair competition for formal businesses (B2; P1).

An interesting factor that can be discussed here and linked to *self* is the formal versus informal economy. Whilst those operating in a formalised sector may have access to some financial support, this remains limited. The informal sector may push for CE practices, yet these initiatives are often blocked by the formal sector for fear of competition. This highlights that within a country context; there are often multiple competing forces at play that can either promote or hinder the development of CE transitions. Often these competing forces are linked to economic and, more specifically, monetary factors.

### Drivers

#### Culture, Education and Society

In Peru, efforts are underway to promote not only a CE model, sustainable production and consumption (SDG12), but also decent work (SDG8) (N3). Initiatives such as “Sinba” [57] and “Ciudad Saludable” [58] are contributing to the formalisation of labour in the waste management sector (A4; N1), where approximately 180,000 waste picker families and recyclers still operate informally (N1). This relates to *self*, particularly the capacity to drive change motivated by personal passion and shaped by individual circumstances.

Although citizen awareness has not yet become a decisive factor (Sect. "Culture, Education and Society"), it is beginning to emerge in Perú (P1). Climate change is becoming more familiar to Peruvian society, particularly in the wake of extreme weather events, such as cyclones and high temperatures during the summer (P1). Consequently, Peruvians are increasingly concerned about large industrial projects that pose environmental threats (e.g., the mining sector). Some of these projects have been halted due to social opposition, with communities protesting potential environmental impacts, such as the depletion of water resources (A3). Hence, a generational shift is evident, with younger generations, particularly Generation Z, demonstrating a stronger ecological mindset (N2). Additionally, certain retail practices, such as the promotion of reusable bags, are incentivising consumers to engage with CE practices. The media also reflects this trend, with the CE increasingly being featured as a topic of interest that generates public engagement (A4). Many Peruvians seek to extend the useful life of their belongings due to financial constraints, as the high cost of new items makes them inaccessible (B3). Additionally, a culture of mutual support is prevalent, especially in smaller communities, where the exchange of goods and services is common (B3).

Several *strategies* are implemented that can support the transition, especially through creating increased awareness of the CE concept in society. The more society is engaged in the process, the more supportive the *situation* becomes to transition towards a CE.

#### Policy and Regulation

There is political will to advance the CE in Peru, “*the issue now is on the table*,* and it is one of the key items on the agenda of the Ministry of the Environment*” (N5). A primary driver of the CE in Peru is the institutional regulatory framework promoting the transition, which is influenced by legislation from Canada, the United States, and especially the European Union (EU) (A4). In 2013, the Ministries of Production (PRODUCE for its acronym in Spanish) and Agriculture (MIDAGRI for its acronym in Spanish) began addressing environmental concerns and the CE in their regulations. In 2018, as CE “*could be an important factor to increase the competitiveness of the country and the economic sectors*” (N4), the Ministry of Economy and Finance (MEF) approved the National Competitiveness and Productivity Policy [59], which prioritised promoting environmental sustainability in economic activities (B2, P1). Based on this policy, a series of sectoral CE roadmaps have been developed, including: industry, fisheries and aquaculture, agricultural development and irrigation (pre-published), water and sanitation sector (currently pending approval) (N3, N4), and tourism (under development) (N4). Additionally, Peru is advancing in several legislative measures, evolving from the Integrated Solid Waste Management Law of 2016 [44] to over 60 or 70 CE-related norms (N4). Some examples include: (i) the Plastic Law [40], introduced in response to marine pollution (A3; N1; N4) and (ii) the EPR (N1; P2; N4; N5) included in the solid waste management law [39]. As a result, “*it is much easier for waste to be recovered and to enter into an industrial symbiosis*” (N3). Additional regulations targeting specific products have also been published, such as the tax on plastic bag consumption (P2), a law addressing end-of-life tyres, and packaging regulation (P2).

Another significant driver in Peru is Cleaner Production Agreements (A2), where companies voluntarily commit to achieving environmental targets that surpass existing regulatory requirements. These agreements, which have been particularly effective with large companies as the primary participants (P2), were initially focused on waste management but have since expanded to include environmental indicators such as carbon and water footprints (N5). For many companies, these agreements serve not only as a tool for improving environmental performance but also as a means of market recognition.

Another primary driver of a CE transition in Peru is international cooperation (N4; P1; N3; N4; B2; N2; N5; A1), particularly from the EU (N4; B2; N3). This support extends beyond financial assistance to include capacity building (P3), such as the development of roadmaps (N5). N4 pointed out: “*the first circular economy roadmap for the industrial sector draws heavily from the European Union’s circular economy action plan*”. Consequently, European policies are not limited to internal regulations but extend to their trading partners, requiring that imports comply with environmental standards. This has direct implications for countries like Peru, a significant exporter to the EU (N3). The Peruvian government consistently engages with international cooperation leaders to maintain strong bilateral relations, as “*cooperation has not only been driven by resources but also by strategies that ensure this topic remains at the forefront of the state’s agenda*,* given that the state is the main driver”* (N4).

Additionally, nations in the region, including Chile and Colombia, serve as key influencers and benchmarks for Peru (N3; N5): “*Peru is always looking to Chile when developing regulations*,* as they see the products coming from Chile*,* which meet higher standards and qualities”* (N3). Furthermore, Peru aspires to join the Organisation for Economic Co-operation and Development (OECD) (N5; N3; N1), which requires the adoption of EPR standards and compliance with international environmental and circular economy practices (N1).

To deal with the transition process, there is an active movement towards creating *support* predominantly from international entities wanting to trade with Peru and creating an economic bond. The country is increasingly responsive to fostering these engagements by setting up new entities that can promote circular practices and develop roadmaps to guide different sectors. This case confirms prior findings that government support, political will, and regulatory frameworks are central drivers in Latin America’s CE landscape [8], particularly for advancing recycling and waste management technologies. However, this study also reveals that in Peru, policy momentum is strongly shaped by external pressures, including EU regulations and regional benchmarking with countries like Chile and Colombia.

#### Technology, data, and Research

Research efforts are increasingly focused on implementing CE principles through recycling technologies, waste reuse, and the enhancement of waste management systems in the country (B3). Furthermore, growing financial support for CE research (A2; N5) is encouraging scholars who previously had little engagement with the concept to explore it more actively (A2). Collaboration with international universities is also facilitating a deeper understanding of CE among Peruvian academic institutions (P3). More recently, “*the relationship between academia and business is gaining more strength in the pursuit of tangible outcomes*,* and not just to do a paper*,* which was always the case*” (B1). This growing connection between academia and industry mirrors regional dynamics, where CE innovation is increasingly seen as a tool to enhance resource efficiency and reduce environmental impacts in response to the climate crisis and resource scarcity [60].

#### Economy

In terms of financing a CE, although still insufficient, there are increasingly more funding opportunities available from both private and public sectors compared to previous years (B3). Major financial institutions are developing new products and expanding their finance divisions (P1; B1; N1). Many companies are expressing interest in accessing green funds, which have become increasingly competitive, particularly for small and medium enterprises (SMEs) (B1). For instance, based on a green finance roadmap [61], banks have green financial products, such as lower-interest loans for companies demonstrating sustainable projects (P3), or favourable payment terms (N5). There are also financial products that, while not explicitly labelled as part of a CE, are closely aligned with its principles (N4). One such example is the ‘*Mi Vivienda*’ green bond, which facilitates the purchase of properties that meet specific sustainability certifications, such as efficient use of water and energy, offering lower financing rates compared to conventional buildings (N4). In the public sector, funding is available through programmes such as “Procompite” and “Proinnóvate”, both of which are significant initiatives under the Ministry of Production (N3) to develop training or technical assistance (N4). International cooperation, mainly from the EU, also plays a crucial role in public financing (A2), aiming to incorporate CE-related goals and resources into institutional activities, budgetary programs, and investments (N4).

The CE has gained traction in the Peruvian business landscape through implementing the plastic law (N1) and macro-regional workshops aimed at raising awareness of the CE roadmap (P1), as *“the National Society of Industries and the Lima Chamber of Commerce are already starting to identify the circular economy as an opportunity*,* not just a trend*” (N4). The tourism and housing sectors have expressed interest in aligning with the Peruvian CE roadmap, and trade unions in the fishing and industrial sectors are advocating for the transition, “*because they understand that this helps them”* (P3). Furthermore, given Peru’s status as one of the most biodiverse countries globally (N1), there is a growing belief that the country is at a pivotal moment to embrace the CE, through the development of bio-based and green businesses (N1; N3), focused on how the circular model can support bio-loop initiatives, such as reforestation and ecological restoration efforts (N1); where regenerative agriculture is gradually emerging in the Peruvian market, primarily led by multinational corporations (N1). Large international companies are identified as the main motivators of introducing circularity in their operations (A3; N1; N2; N3; P2; N5) because their parent organisations are already adopting these practices (N5). Their motivation is primarily business-oriented, seeking to differentiate from competitors and protect their reputation, rather than being driven by regulatory compliance (P2). Additionally, the cost minimisation by transforming waste into new products, avoiding extracting or demanding virgin raw materials, is another driver identified (B2), as well as increasing resource efficiency (B2), resulting in lower pressure on ecosystems and natural resources (P3).

The external market has played a pivotal role in driving the transition of Peruvian companies towards more sustainable practices (B1; N2; P2; N5; N1; A3). Peru is a significant supplier of resources related to the agricultural and fishing sectors. As a result, exports have become a key driver for the adoption of environmental measures and regulations (N4; A2; A4), “*because the receiving country requires you to adopt measures*” (A2) as traceability requirements, certifications (e.g. LCA or organic), or recycling packaging (A4). Consequently, Peruvian suppliers are increasingly being asked to implement green and CE practices (B2; N3), a shift impacting not only Peru but also much of Latin America (N3). A notable example of this trend is the adoption of ISO standards by food companies to meet the demands of Free Trade Agreements (FTAs) with the EU and the United States (B2). In response to these external market demands, Peruvian companies are exploring frameworks such as the European Green Deal, climate change initiatives, and the Sustainable Development Goals (SDGs) (B2). This has led to a growing focus on sustainability, CE, carbon and water footprints (B2), and deforestation-free agricultural practices (N3) for export crops.

Concerning incubators, programs and startups, several initiatives are already underway in Peru, with many projects primarily focused on the CE (N3). Co-financing opportunities and funds are available for startups that emphasise innovation, development, and research, including programmes such as ProCiencia and Proinnovate, where CE-related projects are eligible for support (P2). There is a growing interest in this field, with an increasing number of companies actively seeking investors for financial partnerships, technological investment capacity, and other forms of support to advance their CE initiatives (N3).

Overall, there is increasing *support* available to actively engage in a CE. This is predominantly done as a response to external factors, as increasing business and developing the economy in general demand adapting to CE practices, as these are often mandated by external partners. Even though politically Peru may not necessarily be stable and/or going through a lot of change, the transition process seems to be more reliant on economic partners and their demands. This finding complements the literature on Latin America, which identifies potential economic benefits, green market access, and bioeconomy development as key CE drivers [62]. In Peru’s case, however, external market pressures, trade conditions, and international cooperation emerge as unique and particularly strong motivators for transition, a pattern less emphasised in previous studies.

### Strengths

#### Culture, Education and Society

Peru’s biodiversity is a major strength for advancing the circular economy (A4; B3; N2; N4). As A4 states, “*the greatest resource of Peru is not mining*,* it lies in its biological wealth*.” This biodiversity offers renewable natural inputs for multiple products and services that can be reused and recycled across sectors (A2; A4; B2; B3; N2). There is also growing awareness and commitment to sustainably managing this wealth, creating opportunities for Peru to enter emerging circular markets using local inputs and waste materials (N1; B1; P1). Conservation efforts in the Amazon further reveal the country’s potential for integrating nature-based solutions (N1).

Peru’s ancestral cultures maintain a deep respect for nature, often personifying it as sacred (N3; N4). As N4 noted, “*we have cultures with beautiful rituals*,* such as the water festival*,* or the sun festival*,* which recognise nature as a superior being.*” The national CE roadmap [14] includes traditional knowledge, acknowledging the value of centuries-old practices that are inherently circular (A2; N3). As B3 observed, *“you go to a small village and say: here*,* they are already applying circular economy concepts without anyone teaching them.*” In fact, a recent study evaluated circular economy projects in Peru and found that regenerative agriculture inspired by ancestral technologies was the strongest model, followed by bioeconomy and sustainable handicrafts [63]. These results confirm that ancestral knowledge is not just cultural heritage, but a living, applicable form of circular technology.

Peru also has a strong culture of reuse and repair, particularly in response to limited financial resources (A1; A3; B3; N2; N5; P2). Common practices include repairing shoes, electronics, textiles, and cars, with widespread access to skilled technicians (N2). “*Reusing is a cultural issue; we do it a lot and unconsciously*” (B1). Informal markets like *cachina*s exemplify this dynamic, where goods are repaired, repurposed, and resold (A2; B1; P1; P2). Finally, ingenuity and creativity are seen as defining features of Peruvian society (B2; N1; N2; P1). Resourcefulness thrives in contexts of scarcity, as shown by second-hand dealers, scrap collectors, and informal recyclers who already apply circular practices (N2). *“In Peru*,* we are very creative… when it comes to solving problems*,* we find a way*” (P1). Despite their informality, many small businesses embody resilience and innovation (B2; N1; N4). As N1 puts it, “*the Peruvian entrepreneur is*,* by essence*,* a survivor*,* resilient and ingenious.*”

Peru’s rich biodiversity and strong ancestral ties to nature create a favourable *situation* for CE adoption, providing a foundation for regenerative practices and nature-based solutions. The *self-*dimension is reflected in the resilience, creativity, and adaptive behaviours of both individuals and enterprises.

#### Policy and Regulation

Peru has made significant progress in consolidating a favourable framework for this transition (N3). First of all, a CE coalition that includes the National Society of Industries, various companies, state actors, and universities, forming a multi-stakeholder platform at the national level, have started to be formed (A4; P1).

*The national circular economy coalition is expected to serve as a stable space where key actors remain engaged and provide leadership regardless of changes in government. In the future*,* the coalition will become a non-profit legal entity*,* which means it will have a legal personality and a legal representative who will operate independently of political shifts* (N4).

This multi-stakeholder space, which began as a platform, is now being formalised as a non-profit legal entity, which will allow resources to be leveraged and mobilised, fostering a greater understanding and adoption of the CE in the country (N3; N4).

Another strength of Peru is the CE roadmap [44], which is considered an asset since it has been integrated into the national policy on competitiveness and productivity since 2018, incorporating sustainability as a priority objective (A2; N4; N5; P1; P3). The roadmap document is cross-sectoral and is managed by different ministries, with a horizon towards 2030 (A2). It is estimated that by 2030, more than 750 companies will be applying circular economy practices, increasing employment and raising the national gross domestic product by 2% [64]. These instruments provide regulatory *support* for CE adoption.

#### Technology, Research and Data

The Business Technological Innovation Centres (CITEs, for its acronym in Spanish), of which there are over 40 in Peru, prioritised by regions and value chains [65], play a crucial role in providing technical assistance, reducing technological gaps and enhancing the added value of products through technological and procedural innovations (N3). Despite limited resources, these centres have made significant advancements, and about 10% of their consulting services focus on CE, a percentage that continues growing (N4). Additionally, initiatives like the CREEAS platform (Regional Committee of Business, State, Academia, and Civil Society) in the region of La Libertad [66], which integrates universities, CITEs, civil society organisations, and the government, demonstrate the potential for multi-actor collaboration to capture and lead innovation projects sustainably (N3).

The development of eco-industrial parks in Peru, particularly the five well-established ones in Lima, represents a significant strength for the transition to a CE. These parks utilise technological advancements, data centralisation, and Industry 4.0 to optimise sustainability and efficiency in their operations (P3). The involvement of universities, startups, and entrepreneurs in CE projects shows an active foundation for generating ideas and innovative business models, integrating material technologies and components of bio and eco businesses with circularity (N3).

#### Economy

The empowerment of the industrial sector, both in MSMEs and large companies, is a key strength for the transition to a CE in Peru. Several MSMEs have shown some progress in adopting CE principles, leveraging their ability to be more agile and adaptive. “*The adoption of circular economy principles seems to be more concentrated in medium and small enterprises*” (A2). These emerging businesses not only demonstrate a different approach to sustainability but also incorporate reuse and repair practices as part of their business model (A2). Innovative business models can leverage existing practices and opportunities to drive systemic change. For example, the practice of refilling bottles instead of selling new plastic ones avoids plastic disposal and reduces microplastics, which is particularly relevant in regions like Iquitos (N1). Similarly, leasing-based models, such as those applied to computers and printers, also demonstrate how circularity can emerge from service-oriented logic, shifting the focus from ownership to access and maintenance. These approaches, already normalised in many sectors, lay a cultural and infrastructural foundation for scaling circular business models across industries (P1).

In parallel, large industries in Peru have also begun to show a significant commitment to the CE, a shift in perception that was unthinkable a few years ago. Indeed, “*in the last 2 or 3 years*,* it has been discussed with great conviction by the National Society of Industries*” (P1). Large companies in sectors such as extractive and fishing are regulated and controlled, and have started to adopt eco-efficiency guidelines. “*The existing technology is in the hands of major industries*” (A4), enabling these companies to make significant changes towards sustainability. The National Fisheries Society, for example, has working groups dedicated to sustainability that address the carbon footprint and life cycle assessment (B1). This strategic approach and access to greater financial and technological resources have allowed large industries to play a crucial role in the transition. Thus, *strategy* is visible in the diverse implementation pathways: from sector-specific approaches like eco-industrial parks and reverse logistics, to business model innovation in both MSMEs and large industries. While these strategies vary in scope and maturity, they collectively illustrate a shift from isolated efforts toward systemic change.

### Key Stakeholders

**Fig. 1:**
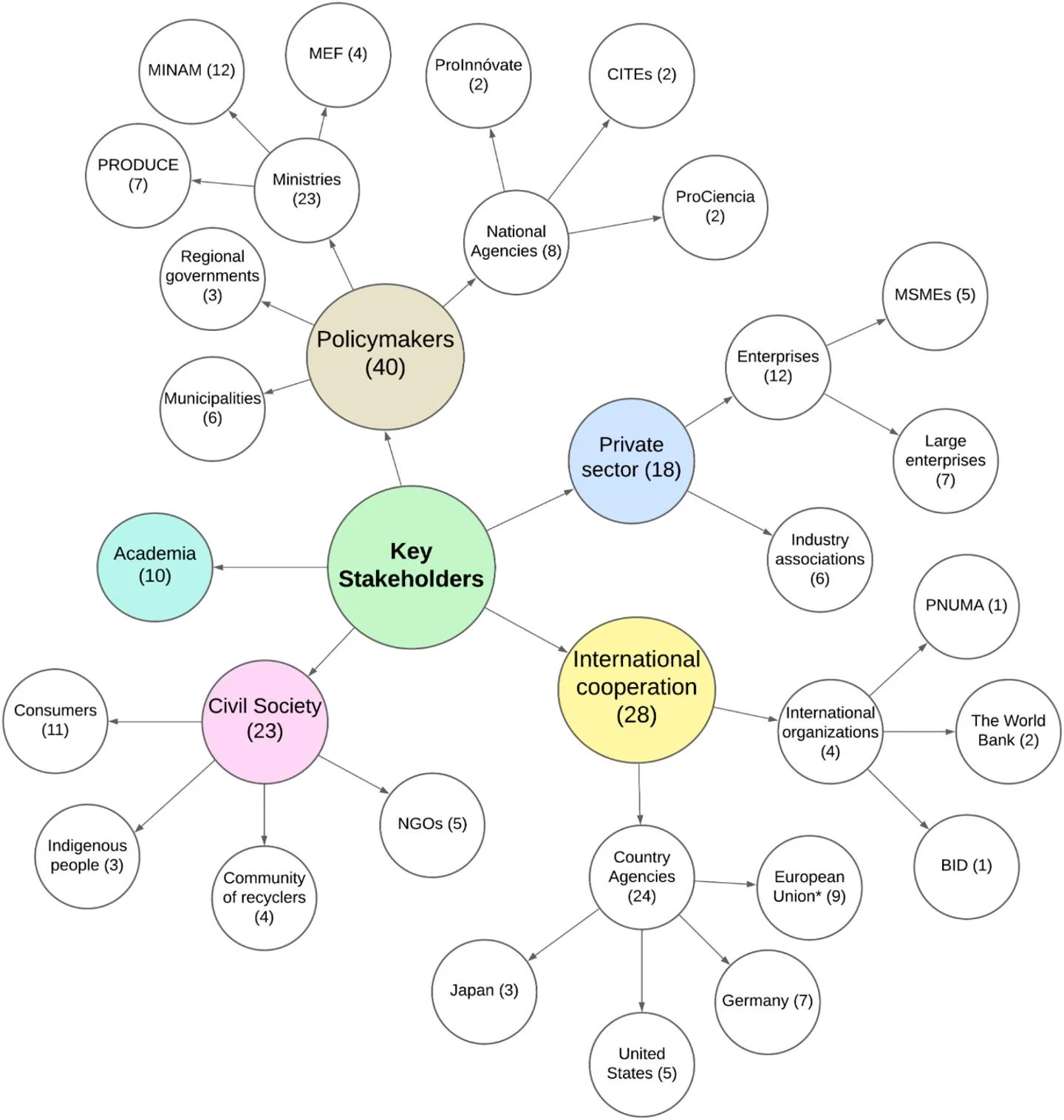
Key stakeholders identified in the interviews. The numbers next to each stakeholder represent how often they were mentioned by interviewees, with the size of the coloured circles also corresponding to this frequency. MINAM: Ministry of Environment, MEF: Ministry of Economy and Finance, PRODUCE: Ministry of Production, CITEs: Business Technological Innovation Centres, ProCiencia: National Program for Scientific Research and Advanced Studies, ProInnóvate: National Program for Technological Development and Innovation, MSMEs: Small and medium-sized enterprises, NGOs: Non-governmental organisations

Based on the responses of the interviewees, the analysis of stakeholders was divided into five main groups: civil society, academia, policymakers, private sector and international cooperation (Fig. 1).

#### Policymakers

Most interviewees identified the central government as the main stakeholder. Within this group, the importance of the Ministry of Environment (MINAM) is highlighted as crucial, as it is the entity in charge of establishing the institutional and regulatory framework for the adoption of CE practices in Peru (A3; A4; N1; P2). As mentioned in the previous sections, MINAM has prioritised CE, evident in the national CE roadmap and the national coalition [49], though political instability may affect its actions (N4; N5; P1; P3). On the other hand, the Ministry of Production (PRODUCE) is also key, linking the government with major economic sectors like manufacturing and mining (N1). In addition, PRODUCE’s initiatives include CE roadmaps for industry and fishing sectors and financial support for CE projects (A4; N3; N4; P3). Due to this, the interviewees consider PRODUCE’s role in the CE Coalition as a chance to boost participation and coordination with other ministries (N3; N4; P1). Concerning this, the Ministry of Finance (MEF) is another important ministry due to its influence in promoting CE-related policies, recognising CE’s potential to enhance sector competitiveness despite some resistance to new regulations (N3; P1). To a lesser extent, the Ministries of Agriculture (B2; N3; P2), Tourism (P1; P2; P3) and Construction (B2; P2) are mentioned as other relevant ministries.

Within local levels of government, the roles of regional governments and municipalities are crucial (A1; B2; N4; N5; P1; P2). Effective coordination between them is important to address centralisation and uneven resource distribution issues (see Sect. "Barriers"), which hinder circularity transitions at local levels (N4; P2). Likewise, their direct consumer contact and ability to create local norms make them essential for this transition (B2; N5). These administrations link informal recyclers with industries capable of processing waste due to their solid waste management role (A1; P1). Additionally, country agencies, which are government entities attached to a ministry, offer financial support and incentives for circularity projects (A1; A4; B2; N2; N3; N4; N5; P2). Programs like ProInnóvate and ProCiencia from the National Council of Science, Technology and Innovation (Concytec in Spanish) are promoting circularity projects by providing loans and competitive funds (N2; P2). Finally, other important entities include the National Institute of Quality, the Informal Property Formalisation Agency and the Development Finance Corporation, which play roles in implementing new technologies and securing CE project financing (A4; B2; N3).

#### Civil Society

Several interviewees highlight the key role of consumers in Peru’s transition to a CE (A1; A4; B1; B2; N2; N5; P1). B1 and N5 agree that consumer awareness of circularity can influence purchasing decisions and increase demand for sustainable products (see Sect. "Culture, Education and Society"). Additionally, waste segregation in households is another aspect that needs to be improved by a sensitised population (A1; N5; P1). “*If the population is not sensitised*,* they will not separate waste at home*,* and that is an issue that needs to be worked on”* (B2).

On the other hand, other interviewees (B1; B3; N1; N4) add NGOs as stakeholders. N1 and B3 highlight the role of NGOs in implementing CE projects through alliances with policymakers and the private sector. NGOs also assist in promoting circularity practices among consumers, increasing interest in CE and sustainability (N1; N4). B3 also emphasises that NGOs’ primary role is to provide funding and resources for citizen-led initiatives. However, there are other organisations, commonly acting as lobbies (B1), that have interests that do not align with the CE, and their impact can be detrimental.

Finally, the community of recyclers, mostly informal, is a subgroup that must be taken into account in the transition process (A1; A3; N1; N2). The Peruvian recycling system depends on their work, and formal integration is vital for CE success, particularly in material-processing industries (A1). Moreover, formalising their knowledge and experience in waste revalorization within the value chain presents an opportunity for the success of CE initiatives (A3; N1; N2). Likewise, indigenous and native communities are also important stakeholders. Their ancient traditions, closely linked to biodiversity, align with modern sustainability and circularity concepts (A2; B3; N4) (see Sect. 4.3.1).

#### Private Sector

Several interviewees agree that large enterprises must be involved in promoting and implementing the transition towards a CE (A1; A3; A4; B1; N2; N3; N5). Their capacity to generate significant changes and innovate in circular technologies and processes is invaluable, reflected in companies like ALICORP or TASA (A3; A4; B1). Currently, large companies in Peru are willing to invest in CE projects because they recognise opportunities in profit margins and market competitiveness (see Sect. "Economy") (A1; P1; P3). Interviewees indicated that companies in the manufacturing (A3), mining (A3; A4; B1; N3), agriculture and fishing (A2; B1; B2; N3; P2) sectors should be the first to prioritise the path towards the transition.

Similarly, some interviewees suggested that the participation of MSMEs is equally important (A2; B2; N2; N5; P2). MSMEs often face obstacles like informal status, lack of interest and financing, affecting their participation in the transition to a CE (A2; N2; N5; P2). Similarly, business associations, which unite various companies, are also significant due to their influence on promoting CE practices among large and small companies (A4; B1; B2; N1; P2; P3), as well as facilitating communication and collaboration with the government and other stakeholders (A4; P2). This has been reflected in the National CE Coalition, where both government and private sector actors participate. The main unions mentioned are the National Society of Industries, the National Fisheries Society, the Association of Exporters, and the Chambers of Commerce (A4; N3; P2; P3).

#### International Cooperation

A large majority indicates that international institutions are crucial in Peru’s CE transition. Most interviewees emphasise the extensive financing offered by the European Union, directly or through its countries’ international cooperation agencies. The development of private sector pilot projects, the creation of multi-actor platforms with the government, and academic collaborations (A2; B3; N5) reflect this vital connection. The German Agency for International Cooperation (in German GIZ) is specifically noted for its impact on financing and training, creating circular clusters, and providing management training (A1; A4; P1). Other countries like the United States, Japan, Norway, Spain, and South Korea, as well as multilateral institutions like the United Nations Environment Programme (UNEP), the World Bank, and the Inter-American Development Bank, are also mentioned (A1; A2; A4; N3; N4; P1).

#### Academia

Although this topic was not explored in depth, 10 out of 15 interviewees noted that academia plays a role as a stakeholder in the transition towards circularity (see Fig. 1). There is a growing interest in the topic and its implications, although CE research is still in the early stages of development compared to other countries in the region, such as Chile or Colombia (P1; N4). However, some of the interviewees consider that the link of Peruvian Universities with other actors, such as business associations or policymakers, has deteriorated in recent years, affecting the quality of scientific production or the development of projects in conjunction with private companies (A3; N3; P3).

Findings in the literature identified that regional actors and authorities are key stakeholders to foster CE development [67]. Additionally, the transition to a CE requires the formulation of government policies that: (i) set quality standards for materials and the utilisation of recycled materials, and (ii) provide guidance on managing and using this type of waste across the entire value chain [68]. In Peru, a set of legislative measures to address waste management problems has been published, although the follow-up phase to evaluate its success is the challenge ahead. Concerning emerging economies, a lack of recognition and alignment with the market’s particularities (e.g., financial infrastructure and customers’ preferences) and financial inclusion (e.g., a key factor in Mexico) are some obstacles to the adoption of circular models in SMEs [69]. In this sense, they need to consider the national context (e.g., available infrastructure, regulations, or consumer characteristics) for the transition, which requires a systemic perspective from decision-makers and enabling conditions promoted by governments [69]. On the contrary, technological innovations in emerging economies, such as advanced recycling technologies, sustainable packaging solutions, and improved waste sorting and collection methods appear as promising opportunities in the plastic industry [70]. This is a big challenge in the case of Peru, as the country presents deficiencies in technological development. Additionally, to implement sustainable supply chains, Bhalaji et al. (2024) suggest that industries in emerging economies prefer closed-loop supply chain practices, incorporating 6R (Rethink, Refuse, Reduce, Reuse, Recycle, Repair) practices [71]. Therefore, addressing these barriers while leveraging strengths and drivers will be essential for emerging economies to successfully transition to a CE.

#### Key Alignments and Conflicts

It can be appreciated that the main Peruvian ministries (i.e., Environment, Production and Economy) are aligned with international agencies and some actors from the private sector (e.g., large companies). They converge with the idea that CE can enhance competitiveness (e.g., institutionalisation of CE roadmaps) and allows it to meet international market requirements in key sectors such as fishing, agriculture or mining. On the other hand, civil society and academia support the discourse of raising awareness through the dissemination of both new knowledge and the recovery of ancestral knowledge to protect biodiversity and a greater appreciation of marginalized sectors (e.g., informal recyclers).

However, there are some evident differences, being the most critical among the formal and informal sectors. Although most businesses in Peru are informal or actors such as informal recyclers play a crucial role in waste recovery practices, they can pose a disadvantageous competition for formal businesses subject to current laws. Similarly, the implementation of regulations inspired by European models without a clear analysis of the Peruvian reality, including poor oversight, are assimilated as bureaucratic barriers in the private sector, mainly in MSMEs that do not have the monetary capacity to adapt to these regulations. In addition, academia plays a role as a link between companies and the government, but its role is limited in Peru due to the scarce investment and the lack of larger-scale support for the projects they develop. Finally, consumer preference for prioritizing low-cost products poses a challenge for companies to offer sustainable products but with a higher monetary value.

In summary, there is a fragile equilibrium between stakeholder groups due to a clear tendency towards greater conflicts than common goals. The creation of the national CE coalition is an effort to strengthen key objectives and to smooth out differences between them in a space for dialogue, coordination and solution generation. Nevertheless, the success of these platforms will depend on both the political will of policymakers and the efficient integration of informal sectors and alignment with national and international sustainability agendas.

### Priorities

This section outlines the main priorities based on their significance as indicated by the interviewees, categorised into socio-cultural, political, technological, and economic aspects. The percentage in Table 3 reflects the frequency of each topic, considering only the main priorities. Other less frequent priorities are presented in Table S1 in the online resource.

**Table 3:** Main priorities highlighted by interviewees

Aspect	Percentage	Main priorities	ID
Culture, education and society	42%	Raise awareness of the circular economy	A2; A3; B1; B2; B3; N2; N4; N5; P1; P3
		Incorporate the circular economy into education	B1; B2; B3; N2; N4; N5; P1; P3
		Develop capacities in the formal and informal sectors	A2; A4; B2; B3; N4; N5; P2
		Preserve and value environmental knowledge and biodiversity	A3; N1; N3; N4
Policy and regulation	29%	Establish a cross-sectoral and decentralised regulatory framework that includes mechanisms to ensure adherence. Enhance political commitment with a clear definition of responsibilities and enforcement	A1; A2; A3; A4; B1; B2; N2; N1; N3; N4; N5; P1; P2; P3
		Address waste treatment problems, particularly landfills.	A3; B1; B2; N1; N2; N3
Technology and data	19%	Deploy technologies to facilitate the adoption of circular practices.	A1; A2; A3; B1; B3; N3; N4; P1; P3
		Collect, provide, and analyse data to quantify the effectiveness and outcomes of circular economy initiatives.	A2; A4; N4; P1
Economy	10%	Provide financial support and economic incentives for the development of circular business models.	A3; B1; B3; N2; N3; N4; P3

#### Culture, Education and Society

Several interviewees highlighted the urgent need to raise awareness of CE and environmental issues in Peruvian society. Establishing a culture that values sustainability and promotes efficient resource management is crucial (A2; A3; B1; B2; B3; N2; N4; N5; P1; P3). Integrating CE education as a mandatory element at all educational levels is essential to ensure future generations (students in schools and universities, roughly aged 6–22) understand and adopt these principles (B2; N4; N5). N4 remarked, “*it is not just about training on what the CE is*,* but about working on incorporating the CE as a requirement in higher education*”. Developing capacities and awareness from an early age (children aged 3–12, both at home and in school) is critical to embedding sustainable practices as habits (B1; B3; N2; N5; P1; P3).

Priority must also focus on workforce training across the private and public sectors. Public officials, especially at local and regional levels, need training to make informed decisions on CE (A4; B2; B3; N4; N5; P2). Collaborations between the public sector and academia are recommended to educate new generations of young professionals entering the workforce, typically aged 20–30 (A4; B2; N5; P2). Private enterprises must recognise CE implementation as an investment rather than a cost (A2). Training the informal sector is equally vital for efficient resource management (A4; B2). A4 stated, “*Peru is a country that needs to develop*,* professionalise*,* and formalise its sectors*,” adding, “*it is the informal sector that you have to address at the grassroots level… train the informal sector*”.

Addressing social justice and meeting basic needs are essential to enabling sustainable practices, as individuals are unlikely to commit without these being met (A2; N2). Including social justice indicators within circular management is equally important (A2). Furthermore, preserving and valuing traditional Amazonian and Andean knowledge is critical, as this ancestral wisdom can significantly contribute to CE (N3, N4). Rethinking well-being by fostering a sustainable economy that values biodiversity and natural resource conservation is also necessary (A3; N1; N3; N4). “*It is essential to rescue and maintain this ancestral knowledge*,* especially in a context of urbanisation that threatens to lose it*” (N3), highlighting the importance of territorial knowledge (N4).

This finding provides a context-specific insight into Peru’s circular transition: Amazonian and Andean ancestral knowledge is not only culturally valuable but also strategically relevant. While the Agricultural Sector Circular Economy Roadmap (DS 007-2025-MIDAGRI) incorporates these practices, the National Circular Economy Roadmap to 2030 (approved by Ministerial Resolution No. 351-2023-MINAM) includes them only in a limited way. A transversal integration across circular policies is needed, given the potential of these knowledge systems to support locally driven innovation and territorial resilience. This perspective offers a conceptual contribution that remains underexplored in regional CE literature.

#### Policy and Regulation

Standards, laws, policies, and decentralised mechanisms aligned with CE principles must be created to transition to a CE in Peru (A3; A4; B1; B2; N1; N2; N3; N4; P1). The governance structure must adapt to the national context, considering the specific needs of both the capital and provinces (P1). Regulations for waste and pollution reduction are a priority, as they could reduce health (N1; N5; A2) and environmental problems and offer opportunities for citizens and businesses. Many informal landfills do not meet waste management standards, and even formal ones lack adequate infrastructure (A3; B1; B2; N1; N2; N3).

Another challenge is the lack of enforcement, requiring responsible agencies with clear roles and enforcement procedures to protect the public interest (A1; B2; N3; N5). N5 states, “*the single-use plastics regulation from 2020 has yet to see the release of technical guidelines for enforcement. Although it is in effect*,* enforcement is lacking due to the absence of a clear methodology*”. This highlights the need for a formal government commitment to advance the CE with specific goals and timelines (A2; A3; P3). A2 adds, “*while we have a roadmap as part of national policy*,* there is no obligation for companies to adapt.*” As recent studies emphasise, circular economy roadmaps in Latin America often lack binding mechanisms and incentives, which reduces their transformative potential [72, 73]. The regulatory framework should involve government, private sector, academia, international organisations, and civil society (N4; P1; P2). N4 notes, “*One of the major challenges is achieving coordination among stakeholders*,* and more importantly*,* ensuring they work together*”. Integrating the informal sector into CE initiatives is essential for recycling and circularity (A1).

#### Technology and Data

Due to Peru’s waste management crisis, it is essential to equip infrastructure with technologies to enhance waste collection and treatment, including a decentralised logistics framework for economic viability (A2; A3; B1; N3). As A3 notes, “*investment in infrastructure should extend beyond landfills; such infrastructure must be accompanied by technologies for waste utilisation and improved waste separation*”.

P1 suggests, “*I would improve the roadmap by providing clearer indicators*,* because one of the measures is the number of circular economy regulations*,* which is not an impact indicator.*” This observation (P1; A2; A4) highlights the need to prioritise outcome-oriented metrics to monitor actual progress toward circularity. Although the roadmap acknowledges the absence of indicators such as Peru’s circularity rate (A2; A4), it lacks concrete mechanisms to address this. Based on these insights, this study contributes by emphasising the importance of measurable and decision-relevant indicators. As recent literature notes, without binding metrics and solid data systems, CE strategies risk remaining normative rather than driving systemic change [72, 73].

#### Economy

Promoting financial investment and economic incentives is a priority to advance CE adoption in Peru (B1; B3; N4), particularly due to the prevalence of micro and small enterprises and the lack of resources for sustainable practices. Many still rely on traditional economic models. N4 states, “*we need to work significantly on investment… if I don’t find an incentive*,* I’ll stick with the conventional*”. Thus, improving communication strategies to highlight both the environmental and economic benefits of circular models is essential to increase profitability and capitalise on market opportunities (A3; B1; P2).

Incorporating the informal sector, which represents 74% of employment in Peru, is crucial. As N2 stated, “*the circular economy can be driven by business models that integrate informality and innovation*,* leveraging the ingenuity of the population to create value from waste.*” P3 added that formalisation is a priority to access incentives and funding. Although the national roadmap mentions inclusion, it provides no clear guidance on how to achieve it. The connection between these insights and proposals such as functional formalisation points to a relevant path for high-informality contexts like Peru, where integrating informal actors into the circular transition is both a necessity and a structural opportunity. This reflects broader debates in emerging economies, where informality is recognised as a structural dimension of CE transitions and its integration is key to socially just pathways [19].

## Circular Economy in the Global South and Emerging Economies

In LAC, cultural [18, 49, 50] and financial constraints [49, 74], limited awareness and low education level [49], as well as the lack of regulations and policies on solid waste management [8, 18], have been barriers identified in the literature. A similar trend is observed in emerging economies. For instance, the lack of strong regulatory initiatives in India [75] and Bangladesh [76]; financial constraints and lack of awareness in India [77]; the lack of public training programs to raise environmental awareness in Bangladesh [76]; the relative lack of regulation to protect workers’ health in low and middle income countries [78], and the resistance to change, as well as the lack of expertise, technical and technological capacity, industrial support and supply chain integration in Pakistan [79, 80]. Although similar limitations have been observed in Peru, additional barriers have been identified, including challenges faced by MSMEs regarding circular practices, a lack of coordination among government and private stakeholders, and the presence of resource-intensive industries.

Drivers such as governmental support and political influence in waste management [8], commitment to international environmental agreements, and the impact of global commerce [8] are motivating the transition in LAC. The above is aligned with the case of Peru, although factors such as the formalisation of labour in the waste sector, the pressure of international markets, and the role of transnational companies in the country are new enablers in the region. Additionally, the main enabler for emerging economies is the reduction of costs related to raw materials, energy, and other resources [79–81]. Other drivers mentioned in the literature include enhancing competitiveness, incorporating environmental considerations into core business operations [79]; increasing international competition and sustainability regulations [80]; and the transparency along the supply chain and the availability of circular data [81].

Opportunities in the LAC region for implementing circular models are business innovation in waste management and new energy sources [82], economic diversification, and the development of sustainable businesses, products, and employment [8]. Additionally, opportunities in emerging economies for improved environmental health and boost an intersectoral approach in low- and middle-income countries [78], represents a win-win option from economic perspectives in Sri Lanka [76], and it facilitates the benefits of reuse and recycling waste and further reduces greenhouse gas emissions in India [75], increased potential for job creation, innovation, and resource productivity [83]. Although no opportunities were identified in the Peruvian context within this research, the literature highlights multiple opportunities, such as the use of biodegradable waste for bioenergy purposes [84]; the promotion of food waste collection for composting among Peruvian smallholder farmers [85]; the integration of CE in SMEs [86]-although this study observe that small companies perceive circular practices as costly; suitable wastewater treatment for eutrophication mitigation and indirect potable reuse [87]; circularity norms and actions in food waste management [88]; and the use of machine learning to predict municipal solid waste generation at the household level [89].

The recent bibliometric review of CE in emerging economies by Uwuigbe et al. (2025) suggests that strengthening interdisciplinary collaboration can improve CE research and emphasises the need for expanded global cooperation in emerging economies [90]. In addition, the literature review by Ndoka et al. (2025) identified financial and technological limitations, regulatory and institutional challenges, and cultural resistance, among others [91]. In contrast, policy support, innovation, stakeholder engagement, and capacity building are enablers in developing economies, which are similar obstacles and drivers identified in Peru. Additionally, transformative principles for CE transitions in the Global South were proposed by Hadfield et al. (2025)., such as local economic development, cultivating collaborative action, and regenerating natural systems, among others [92]. These can be complemented with data analysis to quantify the effectiveness of circular initiatives, as was prioritized in Peru, and can be replicated in similar countries.

## Conclusions

This research provides, through the analysis of 15 semi-structured interviews, the first comprehensive analysis of the barriers, drivers, strengths, stakeholders and specific priorities to the transition to a circular economy in Peru, an emerging economy in Latin America. This journey reflects broader challenges faced by the Global South, including economic, social, and political dynamics, while highlighting the unique strengths and priorities of the Peruvian context.

Another major novelty lies on being one of the first articles to apply Schlossberg’s theory, which originates in work psychology, to a country context to fully understand transitioning, here, towards a circular economy. Within the article, we not only demonstrate a contextual contribution by applying the theory to a country rather than an individual (human) context, but also demonstrate that the 4Ss framework can be applied to explain the CE transition. Within a country context, the 4Ss framework is complex as multiple of the Ss are interacting simultaneously to either make the change smooth or act as a hindrance. Within this context, it further became apparent that *support* is vital in driving change, whether this is from the government or at the company level. Without *support*, new baselines to foster a transition cannot be shaped. The *self*-further can be expanded to include sectors as a whole that may act in uniformity, for example, the informal sector organised itself to drive the CE forward, whilst this was seen to be a threat to the formal sector. We can also see a power dynamic emerging between the individual components of the 4Ss framework, which was out of scope for this research, but should be investigated further in the future.

The transition to a circular economy in Peru faces significant barriers, including limited consumer awareness of circular economy principles, financial constraints for micro, small, and medium enterprises, weak enforcement of circular economy-related laws, and technological deficiencies. Cultural factors, such as a preference for linear consumption patterns influenced by globalisation, exacerbate these challenges. Furthermore, political instability and fragmented regulatory frameworks hinder the development and implementation of coherent circular economy policies. These barriers emphasise the need for coordinated stakeholder engagement and systemic reforms.

Despite these challenges, Peru exhibits significant drivers and strengths, such as the country’s rich biodiversity and ancestral knowledge for bio-based circular business models and indigenous practices into modern sustainability frameworks. International cooperation, especially from the European Union, serves as a key driver for regulatory advancements, capacity building, and access to funding from major financial institutions. Emerging policies, including sectoral roadmaps and voluntary initiatives such as cleaner production agreements, highlight growing political and private sector engagement. Furthermore, Peru’s cultural resilience, exemplified by traditions of repair, reuse, and resourcefulness, particularly in informal sectors, supports grassroots circularity efforts and demonstrates the potential for community-driven transitions.

The main priorities for a successful transition in Peru include: raising awareness of circular principles and environmental issues, alongside integrating sustainability education at all levels to build long-term habits and capacity for change. Training public officials and the workforce at local and regional levels, including the informal sector, is essential to ensure informed decision-making and efficient resource management. Social justice considerations and the preservation of traditional Amazonian and Andean knowledge further underscore the need for a culturally inclusive approach. In this context, the high prevalence of informality in the Peruvian economy constitutes a structural axis that must be acknowledged, since integrating these actors is indispensable for advancing CE pathways that are socially just, context-sensitive, and sustainable in the long term. On the policy front, the development of clear, enforceable standards and decentralised mechanisms aligned with circular economy principles is paramount, particularly in addressing inadequate waste management infrastructure and fostering collaboration among diverse stakeholders. Technological advancements and improved data collection systems are also necessary to support waste management, enhance logistics, and measure circular economy progress through meaningful metrics. Finally, economic incentives and investment mechanisms must be expanded to encourage adoption, particularly among micro and small enterprises. Formalising the informal sector and integrating innovative business models will be pivotal in achieving a more inclusive and sustainable transition to circularity.

This research, while providing valuable insights into the transition to a circular economy in Peru, has some limitations. First, the reliance on qualitative data from semi-structured interviews may introduce biases related to the perspectives of the selected stakeholders, potentially limiting the generalizability of the findings. In addition, since the interviews were conducted in Spanish, some nuances may have been lost during the transcription into English, even though a back-translation technique was used to avoid meaning loss in the translation process. Second, the reliance on a modest sample of interviews may hinder the ability to contrast the gathered information effectively. Triangulation with policy documents and grey literature helped to mitigate this limitation, yet future research could benefit from expanding the dataset to include additional interviews, survey data, or longitudinal case studies. Third, the study focuses primarily on Peru, and while it provides contextually rich insights, its applicability to other emerging economies may be constrained by differences in cultural, economic, and political landscapes. Additionally, the study does not extensively address the regional variability within Peru, which could influence the adoption and effectiveness of CE practices. Finally, the limited exploration of informal sector dynamics and its integration into formal frameworks leaves room for further investigation into the practical challenges and opportunities of formalising these critical actors. These limitations highlight the need for future research to adopt mixed-method approaches, expand geographical and sectoral focus, and integrate standardised metrics for a more comprehensive understanding of CE transitions.

## Supplementary Information

Below is the link to the electronic supplementary material.


Supplementary Material 1


## Data Availability

The datasets generated and analysed during the current study are not publicly available due to confidentiality agreements with interviewees, but are available from the corresponding author on reasonable request.
