# Supplementary material for: Circular Economy Transition in an Emerging Economy: Current Status and Priorities in Peru
Source: Circ Econ Sustain. 2026 Mar 2;6(2):67. doi: 10.1007/s43615-026-00791-9 (PMC12953262; doi:10.1007/s43615-026-00791-9)
Supplement: Supplementary file 1 — Supplementary Material 1 [file 43615_2026_791_MOESM1_ESM.pdf]

Circular economy transition in an emerging economy of Latin America: Current status and priorities in Peru

Alejandro Gallego-Schmid*[a], Ricardo Rebolledo-Leiva[b], Leonardo Vásquez-Ibarra[c], Alvaro Elorrieta-Mendoza[d], Denisse Milagros Paredes Cotohuanca[e], Claudia E. Henninger[f], Ana Belén Guerrero[g,h]

[a] Tyndall Centre for Climate Change Research (Manchester), Department of Civil Engineering and Management, School of Engineering, University of Manchester, Booth Street E, Manchester, M13 9PL, United Kingdom

[b]Centro de Innovación en Ingeniería Aplicada (CIIA), Faculty of Engineering Sciences, Universidad Católica del Maule, Av. San Miguel 3605, Talca, Chile

[c]Department of Computing and Industries, Faculty of Engineering Sciences. Universidad Católica del Maule, Av. San Miguel 3605, Talca, Chile.

[d]Peruvian Life Cycle Assessment & Industrial Ecology Network (PELCAN), Departamento de Ingeniería, Pontificia Universidad Católica del Perú, Av. Universitaria 1801, San Miguel 15074, Lima, Peru

[e]Department of Engineering, Pontificia Universidad Católica del Perú, Av. Universitaria 1801, San Miguel 15088, Lima, Peru.

[f]Department of Materials, School of Natural Sciences, University of Manchester, Booth Street E, Manchester, M13 9PL, United Kingdom

[g]Instituto de Investigación en Ciencias Naturales y Tecnología (IARNA), Universidad Rafael Landívar. Vista Hermosa III, zona 16 Ciudad de Guatemala, Guatemala.

[h]Trisquel Consulting Group. Quito, Ecuador.

*Corresponding author: alejandro.gallegoschmid@manchester.ac.uk

Interview questionnaire

Introduction

1. How would you describe your level of knowledge of the circular economy concept (very high, high, medium, low, very low)? How would you define the circular economy?

Barriers, opportunities, drivers and benefits of circular economy

2. What are the main barriers and weaknesses to promote and expand the circular economy in Peru? What factors may frustrate/discourage the transition to the circular economy in Peru?

3. What are the main opportunities and strengths associated with the circular economy in Peru?

4. What are the main drivers of the circular economy in Peru?

5. What are the main potential benefits of the circular economy in Peru?

Specific topics: Policies, finance, technologies, international trade, education, business models, research and key stakeholders

6. What are the main current policies available to support a transition to the circular economy in Peru? Are they sufficient? Are they implemented correctly? What else is needed?
7. What is the current level (very high, high, medium, low, very low) of financial support for the transition to a circular economy in Peru? Is it sufficient? What is needed? What are the most important sources of financing for the circular economy in Peru? What should be the priority areas for circular economy financing?
8. What is the current level (very high, high, medium, low, very low) of technological support for the transition to a circular economy in Peru? What is the role of Industry 4.0 and artificial intelligence? Is it sufficient? What is needed?
9. What is the role (very high, high, medium, low, very low) of international trade in the transition to a circular economy in Peru? What needs to be changed?
10. What is the current level (very high, high, medium, low, very low) of knowledge about the circular economy in Peru? What aspects should be covered in training and education? What should be the priority?
11. What is the current level (very high, high, medium, low, very low) of development of circular business models in Peru? Do you have examples? What types of circular economy projects or companies attract little or no funding? Why?
12. What is the current level (very high, high, medium, low, very low) of research and knowledge transfer on the circular economy in Peru?
13. Who are the key actors capable of accelerating the transition to the Circular Economy in Peru? (Politicians, Private Companies, Citizens/Consumers, Public Administration, Science and Technology Institutions, ...)

Implementation of circular economy

14. What is the current level of implementation (very high, high, medium, low, very low) of the circular economy in Peru? How do you expect it to evolve in the next five years?
15. Can you mention any successful examples of the implementation of the circular economy in Peru? Are there current circular economy practices in Peru that can be applied elsewhere worldwide? What practices from other parts of the world could be applied in Peru?
16. In which specific sectors do you think a transition to the circular economy would benefit Peru the most? What challenges would they face?
17. What other actions would you recommend to facilitate a transition to a circular economy in Peru? What should be the priorities?
18. How would you define a successful circular economy in Peru?

Final thoughts

19. Is there anything else you would like to say about the circular economy or about what we have discussed? Do you have any other reflections or additional recommendations?

20. Would you recommend someone else for this interview? Who should they speak to before returning to the United Kingdom? Any place to visit?

Table S1: Main priorities reported by interviewees (each letter represents one sector. A: Academia, B: Business, C: Consumers, N: Non-governmental Organizations, P: Policymakers).

Interviewees	A1	A2	A3	A4	B1	B2	B3	N1	N2	N3	N4	N5	P1	P2	P3	Total
**Education**																
Incorporate the circular economy into education					1	1	1		1		1	1	1		1	**8**
Develop capacities in the formal and informal workforce		1		1		1	1				1	1		1		**7**
Access to Practical Tools for Circular Economy				1												1
Promoting Circular Economy among Youth, Considering their Informational Context and Prior Awareness													1			1
**Culture and society**																
Raise awareness of the circular economy		1	1		1	1	1		1		1	1	1		1	**10**
Preserve and value environmental knowledge and biodiversity				1				1		1	1					**4**
Promoting a culture of repair and maintenance	1		1	1												3
Business Formalization as the Foundation for a Transition Towards a Circular Economy Model				1					1							2
Territorial Analysis of Peru for the Identification of Context-Specific Needs and Experiences											1					1
**Policy and regulation**																
Establish a cross-sectoral and decentralised regulatory framework that includes mechanisms to ensure adherence. Enhance political commitment with a clear definition of responsibilities, and enforcement	1	1	1	1	1	1		1	1	1	1	1	1	1	1	**14**
Address waste treatment problems, particularly landfills			1		1	1		1	1	1						**6**
Integrating the informal sector into CE initiatives	1															1
Declare climate change as a national priority									1							1
Expanding policies on renewable energy and reducing subsidies for fossil fuels										1						1
Incorporating environmental conditions into public procurement													1			1
Promote the use of environmental labels													1			1
Implementing Extended Producer Responsibility (EPR) in large organizations to ensure sustainable lifecycle management			1													1

Monitor the adherence to deforestation-related regulations and implement complementary measures to reduce its impact								1								1
**Technology**																
Deploy technologies to facilitate the adoption of circular practices	1	1	1		1		1			1	1		1		1	**9**
Collect, provide, and analyse data to quantify the effectiveness and outcomes of circular economy initiatives		1		1							1		1			**4**
Optimize waste separation at the source to improve treatment efficiency and impact								1		1						2
Align environmental indicators with the principles of social justice		1														1
**Economy**																
Provide financial support and economic incentives for the development of circular business models			1		1		1		1	1	1				1	**7**
Develop circular business models		1											1			2
